# Utility of preoperative electrophysiological testing of the facial nerve in patients with vestibular schwannoma

**DOI:** 10.1371/journal.pone.0226607

**Published:** 2019-12-23

**Authors:** Przemysław Kunert, Anna Podgórska, Tomasz Andrzej Dziedzic, Andrzej Marchel

**Affiliations:** Department of Neurosurgery, Medical University of Warsaw, Warsaw, Poland; Faculty of Medicine, Cairo University, EGYPT

## Abstract

**Purpose:**

The aim of this study was to analyze the utility of various preoperative electrophysiological tests of the facial nerve CNVII in predicting CNVII function after vestibular schwannoma surgery.

**Methods:**

This retrospective study included 176 patients who had preoperative facial nerve electroneurography and electrically evoked blink reflex testing. We defined the following variables: axonal degeneration of CNVII (AD-CNVII), trigeminal nerve damage (D-CNV), disturbances in the short-latency pathway of the blink reflex (D-BR), and any changes in electrophysiological test results (A-EMG).

**Results:**

AD-CNVII, D-CNV, D-BR, and A-EMG were noted in 24%, 10%, 64%, and 71% of the patients, respectively. Negative D-CNV correlated with good CNVII function in early (p = 0.005) and long-term follow-up (p = 0.003) but was not an independent prognostic factor for postoperative facial muscles function. D-CNV appeared to be closely related to tumor size. D-BR was related to tumor size and had no predictive value. AD-CNVII (amplitude reduction of 50% or more compared to the healthy side) was an independent factor associated with increased risk of facial muscles weakness (p = 0.015 and p = 0.031 for early and late outcomes, respectively).

**Conclusions:**

Further studies are needed to establish which tests and cut-off values are the most useful for predicting post-surgical facial nerve function.

## Introduction

Intraoperative electrophysiological monitoring of the facial nerve (CNVII) in vestibular schwannoma surgery (VS) is critical for preserving its function [1]. Specifically, intraoperative stimulation thresholds obtained during surgery but after tumor removal have predictive value for postoperative CNVII function [2, 3]. However, outcome prediction using test results obtained prior to surgery is not yet possible, and the relevance of preoperative neurophysiological evaluation of CNVII function is not clear. For example, Darrouzet et al. report that the changes in the preoperative blink reflex test correlate perfectly with difficulties in CNVII dissection and with postoperative facial muscles weakness [4]. However, van Dinther et al. concluded that postoperative CNVII function cannot be predicted based on pre-operative neurophysiological testing [5].

The disadvantage of preoperative electroneurography of CNVII is that we can examine only a portion of the nerve, and that portion is distal to the site of potential damage. Therefore, extending the electrophysiological evaluation by using the blink reflex, facial muscle response potentials evoked by transcranial magnetic stimulation, or needle electromyography of the facial muscles could reveal the real extent of facial nerve and brain stem damage at the site of tumor compression.

The aim of this study was to analyze the components of electrophysiological tests that were performed in the preoperative period and predictive value of neurophysiological abnormalities on the preservation of CNVII function after VS surgery. The size of the tumor is considered the most important factor in terms of preserving facial nerve function [6, 7]. Our series involved primarily large tumors (grade T4 on the Samii scale) in order to identify factors other than size that predicted the functional outcome.

## Methods

### Patients

The medical files of 197 consecutive patients that underwent surgery at our institution to treat vestibular schwannoma were reviewed retrospectively. Patients with neurofibromatosis type 2 were excluded from the study. Seventeen patients were excluded from the analysis due to a lack of electrophysiological data. Four additional patients were excluded from the study due to other reasons: no follow-up data (n = 2), death shortly after surgery (n = 1), or hypoglossal-facial anastomosis at another center prior to radical tumor removal (n = 1). In the group of 176 included patients, the age range was 18–74 years, the mean age was 46 years, and there were 106 women and 70 men. As many as 68% of the included patients had large tumors (Samii grade T4), and the mean tumor size was 30 mm. The characteristics of the patients are shown in Table 1. The characteristics of the twenty-one excluded patients did not differ significantly from those of the non-excluded patients: age range, 18–74 years; mean age, 49 years; 13 women and 8 men; no paresis of CNVII before surgery, 16/21 (76%); mean tumor size, 32 mm; mean tumor volume, 20 mm^3^. In all cases, the tumor was removed totally via a retrosigmoid approach (“subtotal” or “near-total” removal was not used). Facial nerve function was quantified before and after surgery according to the House-Brackmann grading system (HB) [8]. Early and late outcomes were assessed on the day of discharge and at least 6 months after surgery, respectively.

**Table 1:** Patient characteristics (n = 176).

Gender	
Women	106 (60%)
Men	70 (40%)
Age (years)	
Min	18
Max	74
Mean	46
Symptom duration (months)	
Min	1
Max	360
Mean	46
CNVII function before surgery	
No paresis (HB grade I)	144 (82%)
Any paresis (HB grades II-VI)	32 (18%)
CNV before surgery	
No symptoms	128 (73%)
Any symptom	48 (27%)
*Tumor size (mm)*	
Min	8
Max	72
Mean	30
*Tumor volume (cm*^*3*^*)*	
Min	0.2
Max	66
Mean	14
*Tumor grade*	
Grade I	0 (0%)
Grade II	10 (6%)
Grade IIIA	19 (11%)
Grade IIIB	27 (15%)
Grade IVA	30 (17%)
Grade IVB	90 (51%)
*Pattern of CNVII displacement*	
Anterior	83 (47%)
Anteromedial	57 (32%)
Anterolateral	33 (19%)
Inferior	1 (0.6%)
Inside the tumor	1 (0.6%)
CNVII not identified	1 (0.6%)

The tumor size was assumed to be the maximum extrameatal diameter of the tumor. The tumor volume was calculated according to the formula: 4/3 x 3.14 x A x B x C x 1/8, where A, B, and C refer to the dimensions of the tumor in 3 planes. Tumor grade was classified according to the scale proposed by Samii: grade T1—purely intrameatal, grade T2—intra-extrameatal, grade T3a—filling the cerebellopontine cistern, grade T3b—reaching the brain stem, grade T4a—compressing the brain stem, grade T4b—severely dislocating the brain stem and compressing the fourth ventricle (Matthies and Samii 1997). The facial nerve displacement pattern was classified intraoperatively as follows: anterior, anteromedial, anterolateral, posterior, or inside the tumor. HB—House-Brackmann grading system. CNVII—facial nerve. CNV—trigeminal nerve.

Institutional Review Board approval for the study was obtained (No. AKBE/78/2019).

### Electrophysiological testing

All patients had preoperative facial nerve electroneurography and electrically evoked blink reflex testing. The stimulation was performed using a hand-held bipolar constant-current stimulator with a square-wave pulse of 0.1 ms duration. The facial nerve was stimulated with the cathode placed anterior of the mastoid process with a supramaximal current, and the surface silver-cup recording electrodes were placed over the orbicularis oculi, orbicularis oris and nasalis, or frontalis muscle with the reference electrode at the dorsum of the nose.

The blink reflex was elicited by stimulation of the supraorbital nerve, with the recording electrodes placed over the orbicularis oculi bilaterally and the reference electrode placed at the dorsum of the nose. The stimulation was usually performed using a current of approximately 20 mA; lower or higher currents were used occasionally. To obtain the responses with the shortest latency and the highest amplitude, 8 successive trials were performed with about 7 s intervals. Electrophysiological testing was performed using a Nicolet Viking II or Nicolet Viking IV device.

Direct latency and the amplitude of the muscle response (compound action muscle potential) of the orbicularis oculi, nasalis, and orbicularis oris were measured. The latency of the reflex response R1 and the bilateral R2 component after bilateral stimulation were measured and compared. We defined variables according to the following neurophysiological criteria [9, 10]:

1. Axonal degeneration of CNVII (AD-CNVII): a difference of 50% or more in the amplitudes of the direct facial muscle response of the affected and non-affected sides in at least one facial muscle (= positive AD-CNVII).
2. Trigeminal nerve damage (D-CNV): a delay of 1.2 ms or more of the R1 latency on the affected side compared to the non-affected side or R1 loss and the delay of 7 ms or more of R2 latency bilaterally after stimulation of the affected side compared to stimulation of the non-affected side (= positive D-CNV).
3. Disturbances in the short-latency pathway of the blink reflex (D-BR) fulfilling either of the two criteria listed below:
  - Unilateral R1 loss or a delay of R1 latency of 1.2 ms or more on the affected side compared to the non-affected side.
  - A latency ratio of R1 to the direct response of the orbicularis oculi muscle of 4.2 ms or more on the affected side, (= positive D-BR).
4. Any changes in electrophysiological testing results (A-EMG): abnormalities in 1. 2, or 3 (above).

### Statistical analysis

Four series of statistical analyses were performed:

1. The changes in electrophysiological testing results noted above were correlated with the following quantitative and qualitative factors: patient age; symptom duration; preoperative clinical symptoms of CNV and CNVII disturbances; tumor size, volume and grade; and CNVII displacement pattern.
2. The changes in electrophysiological testing results noted above and other pre- and intraoperative factors were correlated with the following CNVII functions after surgery: anatomical preservation of CNVII continuity during surgery; early outcome; and late outcome.
3. Regression analysis was used to assess whether the electrophysiological changes and other factors were potential independent predictors for CNVII function after surgery (including preservation of CNVII continuity, and early and late outcomes).
4. Specific subgroups were analyzed:
  - Patients with positive AD-CNVII
  - Patients for whom there were intraoperative difficulties with CNVII dissection.

Descriptive statistics were used for data analysis, i.e. number frequencies, percentages, and means and standard deviations. The chi-square test was used to compare the distributions of the results. The significance of differences between the groups was determined by the Mann-Whitney U test. Spearman’s rho correlations and Pearson’s R correlations were used to determine the correlation of variables, and stepwise regression analysis was used to identify independent prognostic factors. Statistical significance was assumed at p < 0.05. The analyses were performed using SPSS 14 PL.

## Results

Although only 32 (18%) of the 176 patients had clinically apparent CNVII paresis before surgery, electrophysiological examination revealed different types of significant disturbances (A-EMG) in 125 (71%). The most frequent pathology was D-BR, which was observed in 64% of patients, followed by AD-CNVII in 24% and D-CNV in 10% (Table 2). When we considered only the group of 144 patients who had no facial muscles weakness before treatment, A-EMG was noted in 98 (68%), D-BR in 88 (61%), AD-CNVII in 30 (21%), and D-CNV in 9 (6%).

**Table 2:** Results of preoperative electrophysiological testing of CNVII and correlations of preoperative electrophysiological testing results with selected factors.

		CNVII before surgery		CNV before surgery		Tumor grade		Tumor size	
	Total	No paresis	Any paresis	No symptoms	Any symptoms	Samii I–III	Samii IV	≤ 30 mm	> 30mm
AD-CNVII (+)	42 (24%)	30 (21%)	12 (38%)	33 (26%)	9 (19%)	18 (32%)	24 (20%)	26 (28%)	16 (20%)
AD-CNVII (-)	134 (76%)	114 (79%)	20 (62%)	95 (74%)	39 (81%)	38 (68%)	96 (80%)	68 (72%)	66 (80%)
D-CNV (+)	17 (10%)	9 (6%)	8 (26%)	7 (6%)	10 (26%)	0 (0%)	17 (15%)	4 (4%)	13 (17%)
D-CNV (-)	155 (88%)	132 (94%)	23 (74%)	119 (94%)	36 (78%)	56 (100%)	99 (85%)	90 (96%)	65 (83%)
D-CNV doubtful*	4 (2%)								
D-BR (+)	112 (64%)	88 (66%)	24 (83%)	80 (66%)	32 (78%)	31 (57%)	81 (74%)	58 (64%)	54 (75%)
D-BR (-)	51 (29%)	46 (34%)	5 (17%)	42 (34%)	9 (22%)	23 (43%)	28 (26%)	33 (36%)	18 (25%)
D-BR doubtful*	13 (7%)								
A-EMG (+)	125 (71%)	98 (68%)	27 (84%)	89 (70%)	36 (75%)	35 (62%)	90 (75%)	64 (68%)	61 (74%)
A-EMG (-)	51 (29%)	46 (32%)	5 (16%)	39 (30%)	12 (25%)	21 (38%)	30 (25%)	30 (32%)	21 (26%)

AD-CNVII = Axonal degeneration of CNVII, D-CNV = Damage of CNV (conduction disturbances of an afferent pattern of blink reflex), D-BR = Conduction disturbance of the short latency pathway of the blink reflex, A-EMG = Abnormal EMG (any notable changes).

*In a few cases, some of the electrophysiological testing results were unclear and were thus excluded from the analysis.

There were 5 patients with visible CNVII paresis prior to surgery but who had no significant pathologies noted in the electrophysiological tests. This represents 16% (5/32) of the patients with paresis and 10% (5/51) of the patients without neurophysiological changes. These patients ranged in age from 47 to 73 years, and they had grade III (n = 1) and IV (n = 4) vestibular schwannomas. The continuity of CNVII was not preserved during surgery in 2 of these cases; in the remaining 3 patients, the late functional outcome was satisfactory. Although re-analysis of the electrophysiological tests showed non-significant amplitude reduction in 3 cases, the two other cases had amplitudes that were similar on both sides, while the D-CNV and D-BR were doubtful.

### Correlation between electrophysiological testing results and different quantitative and qualitative factors

The correlation analysis of preoperative electrophysiological changes with pre- and intraoperative factors showed a correlation of AD-CNVII and facial paresis before surgery (p = 0.03). Positive AD-CNVII was noted in 38% of patients with nerve paresis and in 21% of cases with no paresis. Positive D-CNV correlated significantly with the presence of CNV disturbances (p = 0.002) and was detected in 6% and 26% patients without and with CNV symptoms, respectively. There were significant correlations between positive D-CNV and the degree of preoperative CNVII paresis (p = 0.001), larger tumor size (p = 0.002), greater tumor volume (p = 0.044), and tumor grade (p = 0.001). D-BR was associated with larger tumor size (p = 0.043) and higher tumor grade (p = 0.035), but the relationships were not as strong as the D-CNV associations. None of the pre- or intraoperative factors correlated significantly with A-EMG. None of the electrophysiological variables showed significant correlations with age, gender, symptom duration, or CNVII displacement pattern (Table 3).

**Table 3:** The Spearman rho correlation analysis of electrophysiological changes with pre- and intraoperative factors.

		AD-CNVII	D-CNV	D-BR	A-EMG
Age	rho	.001	.125	.027	-.017
	p	.992	.103	.735	.824
	N	176	172	163	176
Gender	rho	-.074	.019	.049	-.018
	p	.331	.804	.537	.809
	N	176	172	163	176
Symptom duration	rho	-.092	.064	-.001	-.034
	p	.230	.407	.988	.654
	N	173	169	160	173
CNV disturbances of any type	rho	-.073	.240	.117	.054
	p	.333	.002*	.138	.479
	N	176	172	163	176
CNVII paresis before surgery	rho	.167	.253	.142	.145
	p	**.027**	.001*	.071	.055
	N	176	172	163	176
Tumor grade	rho	-.106	.257	.165	.118
	p	.161	.001*	.035*	.120
	N	176	172	163	176
Tumor size	rho	-.041	.237	.159	.137
	p	.586	.002*	.043*	.071
	N	176	172	163	176
Tumor volume	rho	-.024	.15	.070	.070
	p	.749	.044*	.374	.355
	N	176	172	163	176
CNVII displacement pattern	rho	.086	.097	.050	-.019
	p	.259	.208	.526	.799
	N	174	170	161	174

* p < 0.05 –in bold text

### Correlation between electrophysiological testing results, other pre- and intraoperative factors and CNVII function after surgery

CNVII preservation and early and late functional outcome data are presented in Table 4. The correlation analysis of electrophysiological changes and pre- and intraoperative factors with intraoperative CNVII preservation showed a significant correlation of loss of CNVII continuity with variables related to tumor size: higher tumor grade, p = 0.005; larger size, p = 0.004; larger volume, p = 0.000. The risk of CNVII interruption was 18% (16/88) and 3% (3/88) for tumors >10 mm^3^ and < 10 mm^3^, respectively. No electrophysiological variable correlated significantly with facial nerve continuity preservation during surgery. In the positive and negative AD-CNVII subgroups, CNVII interruption was noted in 12% (5/42) and 10% (14/134), respectively.

**Table 4:** Evolution of CNVII function throughout the perioperative period and at late follow-up.

	Before surgery		Continuity preservation of CNVII during surgery	Early outcome		Late outcome	
HB grade I	144 (82%)		Preserved—157 (89%)	13 (7%)	Satisfactory 37%	57 (32%)	Satisfactory 74%
HB grade II	25 (14%)	Any paresis 18%		21 (12%)		45 (26%)	
HB grade III	2 (1%)			32 (18%)		28 (16%)	
HB grade IV	2 (1%)			67 (38%)	Not satisfactory 63%	17 (10%)	Not satisfactory 26%
HB grade V	0 (0%)			22 (13%)		8 (4%)	
HB grade VI	3 (2%)		Not preserved -19 (11%)	21*(12%)		21* (12%)	

Facial nerve (CNVII) function was evaluated according to the House-Brackmann (HB) grading scale. A satisfactory outcome included no or mild paresis until normal symmetry at rest and eyelids could close normally (i.e. HB grades I–III).

*In cases with CNVII interruption during tumor removal, repair procedures such as ‘loco operationis’ or extratemporal anastomosis were performed. However, the results of these procedures were not taken into account in this study. Patients with interrupted facial nerves were assessed at late follow-up as HB grade VI, regardless of whether they had satisfactory anastomosis results, as this could interfere with the evaluation of spontaneous CNVII function recovery.

Similarly, at the early- and long-term assessments, functional outcome was strongly correlated with factors related to tumor size (Tables 5 and 6). However, additional significant correlations were also discovered. Negative D-CNV correlated with good facial nerve function at both the early (p = 0.005) and long-term follow-ups (p = 0.003). Unsatisfactory post-surgical CNVII function was noted in the negative D-CNV subgroup in 58% of patients at the early follow-up and in 24% at the late follow-up. In the positive D-CNV subgroup, an unsatisfactory outcome was found in 94% of patients in the early period and in 53% in the late period. Satisfactory long-term outcome correlated with negative A-EMG (p = 0.037) as well. Furthermore, there was a significant correlation between the pattern of CNVII displacement and its functional outcome at late follow-up (p = 0.02). Satisfactory functional outcome was observed in 81% (67/83), 70% (40/57), and 67% (22/33) of patients with anterior, anteromedial, and anterolateral displacement, respectively.

**Table 5:** Correlation of electrophysiological test results and other variables with unsatisfactory CNVII function at different times.

Facial nerve function				
		Loss of continuity during surgery N° 19	Unsatisfactory early outcome (HB IV–VI) N° 110	Unsatisfactory late outcome (HB IV–VI) N° 46
AD-CNVII /+/ (N° 42)		5/42 (12%)	30/42 (71%)	14/42 (33%)
AD-CNVII /-/ (N° 134)		14/134 (10%)	80/134 (60%)	32/134 (24%)
D-CNV /+/ (N° 17)		2/17 (12%)	16/17 (94%)	9/17 (53%)
D-CNV /-/ (N° 155)		17/155 (11%)	90/155 (58%)	37/155 (24%)
D-BR /+/ (N° 112)		13/112 (12%)	73/112 (65%)	36/112 (32%)
D-BR /-/ (N° 51)		5/51 (10%)	26/51 (51%)	9/51 (18%)
A-EMG /+/ (N° 125)		15/125 (12%)	82/125 (66%)	39/125 (31%)
A-EMG /-/ (N° 51)		4/51 (8%)	28/51 (55%)	7/51 (14%)
Tumor grade I–III (N° 56)		1/56 (2%)	25/56 (45%)	4/56 (7%)
Tumor grade IV (N° 120)		18/120 (15%)	85/120 (71%)	42/120 (35%)
Tumor size ≤ 30 mm (N° 94)		5/94 (5%)	49/94 (52%)	12/94 (13%)
Tumor size > 30 mm (N° 82)		14/82 (17%)	61/82 (74%)	34/82 (41%)
Tumor volume ≤ 10 ml^3^ (N° 88)		3/88 (3%)	45/88 (51%)	11/88 (13%)
Tumor volume > 10 ml^3^ (N° 88)		16/88 (18%)	65/88 (74%)	35/88 (40%)
CNVII Displacement pattern	Anterior	8/83 (10%)	45/83 (54%)	16/83 (19%)
	Anteromedial	4/57 (7%)	44/57 (77%)	17/57 (30%)
	Anterolateral	5/33 (15%)	19/33 (58%)	11/33 (33%)

**Table 6:** Pearson’s R correlations of electrophysiological test results and other variables with CNVII function at different times.

		Facial nerve function		
		Intraoperative continuity Preservation	Early outcome	Late outcome
AD-CNVII	R	-.020	.115	.126
	P	.792	.130	.097
	N	176	176	176
D-CNV	R	-.008	.215	.228
	P	.921	.005*	**.003**
	N	172	172	172
D-BR	R	-.027	.052	.141
	P	.735	.511	.072
	N	163	163	163
A-EMG	R	-.061	.063	.157
	P	.423	.409	**.037**
	N	176	176	176
Tumor grade	R	-.210	.384	.340
	P	**.005**	**.000**	**.000**
	N	176	176	176
Tumor size	R	-.214	.323	.322
	P	**.004**	**.000**	**.000**
	N	176	176	176
Tumor volume	R	-.261	.303	.316
	P	**.000**	**.000**	**.000**
	N	176	176	176
CNVII displacement pattern	R	-.064	.107	.175
	P	.404	.162	**.021**
	N	174	174	174

* p < 0.05–in bold text

### Regression analysis

Three series of multivariate regression analyses were performed for three dependent variables (CNVII continuity preservation and CNVII function in the early and late postoperative periods) to investigate the importance of the same factors as in Part 2. Stepwise logistic regression analysis showed that tumor volume was the only independent factor that affected the risk of intraoperative loss of CNVII continuity (p = 0.003). Linear stepwise regression analysis revealed two independent factors that influenced early- and long-term CNVII function:tumor grade and AD-CNVII. At both early and late follow-up, the impact of the tumor grade was greater than the impact of AD-CNVII: p<0.001 vs. p = 0.015 at the early assessment and p<0.001 vs. p = 0.031 at the long-term assessment, for tumor grade and AD-CNVII, respectively. The remaining electrophysiological parameters, i.e. D-CNV, D-BR, and A-EMG, had negligible value as independent predictors in the evaluated periods (Table 7).

**Table 7:** Regression analysis to assess electrophysiological changes and other factors as potential independent predictors for CNVII function after surgery.

Dependent measure: CVNII continuity preservation. Logistic regression.				
	B	p	95% CI	
Tumor volume	-.045	.003*	.929	.985
A-EMG		.466		
AD-CNVII		.695		
D-CNV		.707		
D-BR		.828		
Tumor grade		.059		
Tumor size		.926		
*Significance of the model*: *F—8*.*57; p—0*.*003; Nagelkerke R*^*2*^*–0*.*107*				
Dependent measure: CVNII function—early outcome. Linear regression.				
	Beta	p	95% CI	
Tumor grade	.403	.000*	.273	.581
AD-CNVII	.180	.015*	.110	1.031
A-EMG	-.023	.770		
D-CNV	.072	.340		
D-BR	-.060	.434		
Tumor size	-.076	.583		
Tumor volume	.066	.481		
*Significance of the model*: *F—16*.*727; p—0*.*0001; R*^*2*^*–0*.*178*				
Dependent measure: CVNII function—late outcome. Linear regression.				
	Beta	p	95% CI	
Tumor grade	.356	.000*	.278	.676
AD-CNVII	.164	.031*	.061	1.251
A-EMG	.055	.500		
D-CNV	.107	.165		
D-BR	.052	.507		
Tumor size	.107	.448		
Tumor volume	.163	.089		
CNVII displacement	.055	.500		
*Significance of the model*: *F—12*.*591; p—0*.*0001; R*^*2*^*–0*.*141*				

* p < 0.05 –in bold text

### Specific subgroups analysis

**a**. Satisfactory CNVII function was observed at late follow-up in 95% (36/38) of patients with small tumors (grades I–III) and negative AD-CNVII. The worst outcomes were observed in patients with grade IV tumors in the positive AD-CNVII group: 50% (12/24) of such patients had grade IV-VI HB paresis. For comparison, an satisfactory outcome was noted in 69% (66/96) of patients with T4 grade tumors and negative AD-CNVII (Table 8). Considering only patients with HB grade I and II paresis, satisfactory results were observed in 33% (8/24) and 53% (51/96) after grade IV tumor removal in the positive and negative AD-CNVII subgroups, respectively.

**Table 8:** Correlation of the electrophysiological variables with long-term outcomes of CNVII function according to tumor grade.

	Grade I–III tumors		Grade IV tumors	
	HB I–III	HB IV–VI	HB I–III	HB IV–VI
AD-CNVII (+)	16 (89%)	2 (11%)	12 (50%)	12 (50%)
AD-CNVII (-)	36 (95%)	2 (5%)	66 (69%)	30 (31%)
D-CNV (+)	0	0	8 (47%)	9 (53%)
D-CNV (-)	52 (93%)	4 (7%)	66 (67%)	33 (33%)
D-BR (+)	27 (87%)	4 (13%)	49 (60%)	32 (40%)
D-BR (-)	23 (100%)	0	19 (68%)	9 (32%)
A-EMG (+)	31 (89%)	4 (11%)	55 (61%)	35 (39%)
A-EMG (-)	21 (100%)	0	23 (77%)	7 (23%)

**b**. There were considerable difficulties with dissection of the CNVII from the tumor during surgery in 22% (38/176) of the cases. In this subgroup, positive AD-CNVII, D-CNV, D-BR, and A-EMG were found in 29% (11/38), 14% (5/37), 72% (26/36), and 76% (29/38), respectively. Among the remaining 138 cases, positive AD-CNVII, D-CNV, D-BR, and A-EMG were noted in 22% (31/138), 9% (12/135), 68% (86/127), and 70% (96/138), respectively; however, the differences in the respective pairs were not statistically significant (chi-square test).

## Discussion

Intraoperative electrophysiological monitoring of the facial nerve during vestibular schwannoma surgery has been common practice for many years. We use it routinely at our clinic and have no doubts about its importance. The predictive value of intraoperative electromyography has been supported by several recent studies [2, 3, 11]. Ideally, we would like to be able to predict the most likely outcome before performing the surgery. However, some studies have concluded that preoperative electrophysiological evaluation does not have prognostic value in terms of postoperative CNVII function [5, 12, 13]. This prompted us to evaluate the prognostic value of our preoperative electrophysiological testing results.

Although the facial nerve is particularly vulnerable to damage, large tumors can also affect surrounding structures such as the brain stem. Thus, one can expect abnormalities not only in CNVII neurography and electromyography, but also in the brain stem reflexes. Such reflexes could help to evaluate the function of the proximal part of the CNVII and other nearby structures. In our study, facial nerve electroneurography and the electrically elicited blink reflex were estimated as part of the routine examination of patients with vestibular schwannoma. These measurements can be repeated and are easy to perform without much discomfort to the patients.

### Research findings and literature review

Normand and Daube noted electrophysiological impairment of the CNVII in 73% of their patients, while only 16% had preoperative facial muscles weakness [14]. Our observations were very similar: 71% of all patients and 68% of those with no clinical CNVII involvement showed significant disturbances in electrophysiological tests. However, only 18% of our cases had visible facial paresis before surgery, indicating that there is frequently subclinical facial nerve involvement. Disturbances in the short latency pathway of the blink reflex were the most common abnormality patterns, affecting approximately 2/3 of the patients. Other series found alterations in the blink reflexes in the vast majority of patients with vestibular schwannoma [15]. For example, Nagahiro et al. observed a delay or absence of the R1 response in 91% of their series [16]. Darrouzet et al. concluded that based on differences in the latency of the R1 responses of the affected and healthy sides, it is possible to predict dissection difficulties and postoperative facial muscles weakness, especially in cases where there is a lack of an ipsilateral R1 or differences exceeding 2 ms [4]. This is not consistent with our observations, but the R1 response was evaluated in a different way in our series. In our analysis, D-BR correlated only with tumor size and grade, and was not considered an independent prognostic factor that influenced the outcome of CNVII function. D-CNV, which is partially related to the R1 assessment, was rarely positive, in contrast with D-BR, but D-CNV showed a strong correlation with tumor size and treatment results. This may indicate that D-CNV has greater prognostic relevance than do disturbances in the short latency response. To our knowledge, the predictive value of such an abnormality has never been described for vestibular schwannoma.

The results of the correlation analysis of the electrophysiological test results with preoperative clinical factors were consistent with our expectations. Positive AD-CNVII correlated significantly with preoperative facial muscles weakness, and positive D-CNV correlated with CNV symptoms prior to surgery. The significant correlations of D-BR and D-CNV with larger tumor size and grade were also not surprising, as they can be explained by greater compression of the brain stem and the proximal portions of the CNV and CNVII by larger tumors [17].

Using electroneurography, Höhmann et al. observed a decrease in amplitude on the affected sides of 65% of patients [18]. Kartush et al. reported a preoperative amplitude reduction in 85% (11/13) of patients [19]. Notably, in that study, each amplitude reduction of 10% or more compared to the healthy side was considered to be significant. In their series, the degree of amplitude attenuation was related to the tumor size but did not predict postoperative facial muscles function [19]. In the series of Prasad et al., 30% of patients had abnormal electroneurography, but the abnormal recording, considered an amplitude reduction greater than 40%, did not correlate with facial nerve function outcome. However, an amplitude reduction of less than 30% predicted good functional outcome (HB grades I–II) in over 80% of patients [20].

We are convinced that the preoperative tests are useful, even though factors related to tumor size are the most significant predictors of postoperative facial nerve function. We showed that an amplitude reduction of at least 50% in the affected side compared to the healthy side (AD-CNVII) was an independent risk factor for an outcome of unsatisfactory CNVII function in early- and long-term follow-up. This confirms that preexisting CNVII axonal damage, even if it is subclinical, is important for setting postoperative expectations. It should be emphasized that electrophysiological testing cannot predict intraoperative difficulties in terms of dissection of the facial nerve from the tumor and loss of CNVII continuity during surgery.

It should be kept in mind during the initial evaluation of the results of preoperative tests that the “any abnomality” parameter, A-EMG, did not prove to be of much value in our study, although it did significantly correlate with long-term facial muscles outcome.

The absence of electrophysiological changes in 5 patients with visible CNVII paresis before surgery was interesting. Re-analysis of the results led us to suspect that the following could have caused this: a partial conduction block, a technical error, or use of too high a threshold to define the amplitude difference as axonal damage. A decrease in amplitude of 50% is commonly accepted in neurophysiology as a significant decrease that reflects axonal degeneration. Furthermore, use of a threshold reduction less than 50% would decrease the specificity of the test. Eventually, a normal muscle response after stimulation in cases with evident paresis led us to suspect that a partial conduction block was present i.e. that there was a lack of conduction in some of the CNVII fibers. Although the axons may not be damaged, a conduction block that is associated with paresis can develop due to demyelination and is reversible. Conduction block may explain the reduction in the R1 amplitude or the lack of R1 response if there is no significant axonal damage.

### Our strategy and the results of treatment

The postoperative results in terms of CNVII function in our series were not as good as those published by the best centers. Our policy was to remove the tumor radically, according to the guidelines established by Samii [21]. Our priority was to achieve a permanent cure, as our patients were mostly middle aged with long life expectancies. In addition, there is a long learning curve for vestibular schwannoma surgery, so we want to note that this series included the first patients who underwent this type of surgery at our institution. On the other hand, for logistical reasons our series included mainly large tumors (mean diameter > 30 mm), since patients with preserved hearing and small tumors are operated on in the Otolaryngology Department.

### Potential application of the research findings

There is a trend away from radical removal in order to decrease the risk of unsatisfactory CNVII function. Currently, preservation of function seems to be a higher priority than complete resection of this benign tumor [7, 22]. Hence, there is a need to evaluate both intra- or postoperative tests, which could have prognostic value for long-term functional outcome. Theoretically, knowing that there is a higher risk of severe paresis after surgery due to preexisting nerve damage could affect important intraoperative decisions. Thus, if neurophysiological changes are noted prior to surgery, especially when dissecting difficulties are encountered during the procedure, near- or subtotal resection may be a more reasonable approach in order to avoid additional trauma to the nerve.

### Drawbacks and limitations of the study

There are several drawbacks and limitations to our study, as well as some strengths. The research was retrospective, and the use of appropriate thresholds for the electrophysiological values requires further evaluation. Notably, however, this is one of the largest series published on this subject. In addition, it is a homogeneous series in which cases were treated the same way in terms of the facial nerve (i.e. no sub- or near-total resection); therefore, the statistical results were not influenced by differences in the aggressiveness of the dissection. It is worth emphasizing that the group consisted mostly of patients with large tumors, so the relationships we detected are especially applicable to these difficult cases. This is particularly important now, since many small tumors are treated by radiosurgery or by otolaryngology teams, with the most challenging lesions being treated by neurosurgeons.

## Conclusions

Preoperative electrophysiological testing in patients with vestibular schwannoma has prognostic value. Axonal damage of the facial nerve in the preoperative period, i.e. an amplitude reduction of 50% or more compared to the healthy side, is an independent factor that increases the risk of facial muscles weakness after vestibular schwannoma surgery. Damage to the afferent pathway of the blink reflex strongly correlated with facial nerve paresis after surgery, but it was not an independent prognostic factor for postoperative facial muscles function. Rather, it appeared to be closely related to tumor size. Using the adopted criteria, disturbances in the short latency response of the blink reflex were frequently related to tumor size and had no predictive value. Thus, further studies are needed to establish which test components are the most useful and what cut-off values should be adopted.
